# Indole-fused benzooxazepines: a new structural class of anticancer agents

**DOI:** 10.4155/fsoa-2016-0079

**Published:** 2017-01-04

**Authors:** Ashok K Singh, Vinit Raj, Amit Rai, Amit K Keshari, Sudipta Saha

**Affiliations:** 1 Department of Pharmaceutical Sciences, Babasaheb Bhimrao Ambedkar University, Vidya Vihar, Rae Bareli Road, Lucknow 226025, India

**Keywords:** Hep-G2 cell lines, indole-fused benzooxazepines, interleukins and caspases, liver cancer, molecular docking and dynamics

## Abstract

**Aim:**

A new series of compounds (**1a–16a**) bearing indole-fused benzooxazepine was synthesized, characterized and evaluated for anticancer activity.

**Materials & methods:**

In this study, all the synthesized compounds were screened via *in vitro* anticancer testing on Hep-G2 cancer cell line. A computational study was carried out on cancer-related targets including IL-2, IL-6, COX-2 Caspase-3 and Caspase-8.

**Results:**

Some of the synthesized compounds effectively controlled the growth of cancerous cells.

**Conclusion:**

The most active compounds – **6a**, **10a**, **13a**, **14a** and **15a** – exemplify notable anticancer profile with GI_50_ <10 μg/ml. Preliminary structure–activity relationship among the tested compounds can produce an assumption that the electronegative groups at phenyl ring attached with indole-fused benzooxazepine are instrumental for the activity. Molecular docking study showed crucial hydrogen bond and π–π stacking interactions, with good ADMET profiling and molecular dynamic simulation.

## Lay abstract

Indole, azepine and six-membered flexible rings are getting much attention for cancer drug discovery. To contribute to the development of drugs for liver cancer, we designed a new structural class of compounds, indole-fused benzooxazepines. All the compounds were subjected to a preliminary bioassay analysis against Hep-G2 cancer cell line to determine their effect. Studies were also performed on various cancer-related targets to understand the mechanism of action. Our findings show that five compounds were of remarkable efficacy and warrant further investigation.

## Graphical abstract

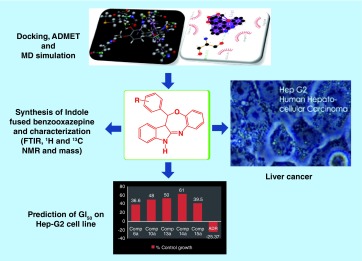

**Figure 1.**
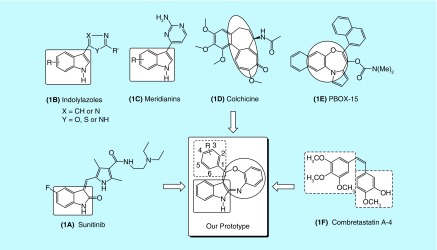
**Rational approach to design phenyl-substituted indole-fused benzooxazepines (1a-16a).**

**Figure 2.**
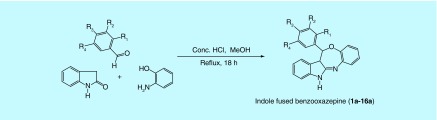
**One-pot three-component-efficient synthetic route to the title compounds (1a–16a).**

**Figure 3.**
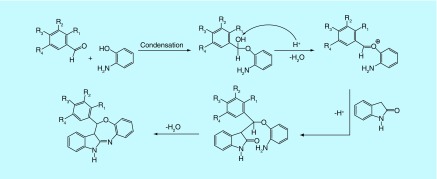
**The plausible reaction mechanism for the title compounds (1a–16a).**

**Figure 4.**
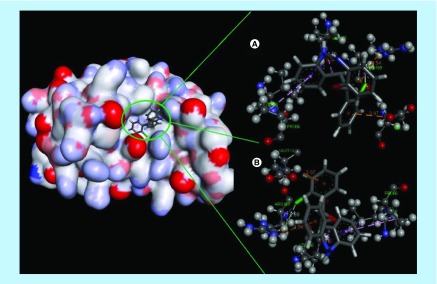
**Docking complex of 14a with IL-6.** **(A)** Structural conformational changes before MD simulation. **(B)** Structural conformational changes after MD simulation: Back bone of active site domain complex, which indicates the contraction of ligand with amino acids residue. MD: Molecular dynamic.

**Figure 5.**
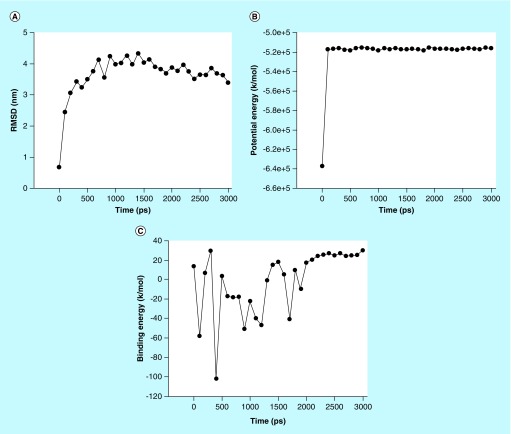
**The stability profile of ligand–protein complex under the molecular dynamic simulation.** **(A)** Average RMSD versus time graph that indicates convergence of the simulated structure toward an equilibrium state with respect to a reference structure (starting structure). **(B)** Potential energy of complex versus time graph that indicates the stability of ligand–protein complex and **(C)** Binding energy of complex versus time graph that also indicates stability of ligand–protein complex. RMSD: Root-mean-square deviation.

Liver cancer is one of the leading causes of cancer-related death worldwide, and accounts for more than 600,000 deaths every year. The majority of patients with liver cancer die within a year after diagnosis [1]. The most common form of liver cancer in adults is hepatocellular carcinoma (HCC), which generally starts as a single tumor that grows larger or as many small cancer nodules throughout the liver. There is currently a demand for the discovery and development of new lead compounds of simple structure, exhibiting excellent antitumor property and new mechanisms of action [2].

Indole scaffold is one of the most widely reputed structural units used to identify new drug candidates as antiproliferative agents. A representative member of this class is sunitinib (Figure 1A), which is currently used in clinics as a multitargeting tyrosine kinase inhibitor for the treatment of renal cell carcinoma and gastrointestinal stromal tumor [3]. Besides this, a diverse variety of indolylazoles (Figure 1B), such as labradorins 1 and 2 and indolylthiazoles, are known for their cytotoxic activities against human lung cancer. Marine indole alkaloids, meridianins (Figure 1C) and their synthetic analogs, have shown prominent anticancer activities against breast cancer [4,5].

The interest in seven-membered heterocycles among synthetic chemists and pharmacologists has increased persistently not only because of the variety of bioactivities but also due to high reactivity and some ubiquitous properties of these compounds. The naturally occurring antimitotic agent colchicine (Figure 1D) and its synthetic analogs have been studied extensively for cancer chemotherapy, however, it lacks *in vivo* anticancer efficacy at its maximum-tolerated dose [6] because its maximum-tolerated dose is limited to around 1 mg/kg [7]. Azepine analogs such as oxazepine have been subject to much investigation, since they are a class of totally synthetic pharmacological agents with diverse action [2,8]. Recently, pyrrolo-1,5-benzoxazepine, a well-known group of microtubule-targeting agents, was shown to display antitumor effects, mainly inducing cell cycle arrest and apoptosis in several human cancer models [9]. A member of this family, pyrrolo-1,5-benzoxazepine-15 (Figure 1E), has shown potent pro-apoptotic activity in a variety of human tumor cell types including liver (Hep-G2), breast (MCF7) and colon (HCT116) cancer cell lines, with minimal toxicity toward normal blood and bone marrow cells [9,10]. A cis-stilbene natural product combretastatin A-4 (Figure 1F) is a lead compound of vascular-disrupting agents targeting tumor blood vessels that binds to the colchicine site and exerts potent cytotoxicity, particularly due to having a cis-configuration-linking bridge and two relatively flexible six-membered hydrophobic rings with the appropriate dihedral angle [11–13].

According to the rational approach of drug designing, the fusion/attachment of relevant heterocyclic rings within a single structural framework can result in a novel scaffold of interest with enhanced biological activity. Thus, inspired by the aforementioned promising findings and the rational approach of drug designing, a novel series of compounds containing an indole moiety directly attached to benzooxazepine with a relatively flexible six-membered hydrophobic ring in a single-molecular framework have been designed and synthesized to establish an important pharmacophoric structure with powerful anticancer potentials. These have further been evaluated for their anticancer activity via *in vitro* screening on the Hep-G2 cell line and molecular docking study with ADMET profiling and molecular dynamic (MD) simulation at Caspase-3, Caspase-8, IL-2, IL-6 and COX-2 receptor site to establish the molecular mechanism of the title compounds. A feasible one-pot-efficient synthetic approach was employed for the synthesis of the proposed derivatives. Some representative chemical structures of important compounds possessing indole, seven-membered ring, benzooxazepine, six-membered flexible ring and our synthesized prototype containing these fragments have been presented in Figure 1, which possibly endows better complementarity with the receptor molecule.

## Materials & methods

### General

The chemicals and reagents were procured from Sigma-Aldrich chemicals and used without further purification. The progress of the reaction was monitored by thin layer chromatography on silica gel G plates using iodine vapors and UV light as visualizing agents. Melting points were determined by open capillary method and are uncorrected. After physical characterization, the compounds were subjected to spectral analysis. The IR spectra were recorded on Perkin Elmer RX1 FTIR spectrophotometer using KBr discs and the values are expressed in per centimeter and only noteworthy absorption levels are listed. The positive mode of ESI-MS spectra was recorded on Waters UPLC TQD mass spectrometer at CDRI, Lucknow. The NMR (^1^H and ^13^C NMR) spectra were recorded at 400 and 100 MHz on a Bruker DRX300 model spectrometer at CDRI, Lucknow. The chemical shifts are reported in parts per million (δ values), using TMS as the internal standard.

### Chemistry

In view of few one-pot synthetic methodologies of azepine moiety using acidic catalysts [14–17], we inspired to prepare our designed compounds by one-pot-efficient synthetic route, which is delineated in Figure 2. By adopting the reported procedures of the Biginelli reaction [18–20], indolin-2-one, an aromatic aldehyde and 2-amino phenol underwent an acid-catalyzed, three-component reaction to constitute a rapid and facile synthesis of indole-fused benzooxazepines (**1a–16a**). The possible mechanism of the reaction is delineated in Figure 3. The first step in the mechanism is believed to be the condensation between the aldehyde and 2-amino phenol. The intermediate so-generated acts as an electrophile for the nucleophilic addition on the methylene group of indolin-2-one, presumably through the formation of enol tautomer. The resulting adduct undergoes condensation between >C = O and NH_2_ to give the cyclized product. The mechanism is somewhat similar to the Biginelli reaction.

Finally, structures of the synthesized compounds were established by IR, ^1^H NMR, ^13^C NMR spectroscopy and MS. The formation of indole-fused benzooxazepine derivatives was supported by the presence of –N = C and >C–O–C< stretching band (1600–1700 and 1200–1300 cm^-1^), and absence of –OH stretching band (3500–3600 cm^-1^) in the IR spectra. In addition, appearance of two azepinic >CH– peaks in aliphatic region (δ = 1.5–4.0) of ^1^H NMR spectra also confirms the formation of oxazepine ring in the reaction. Furthermore, mass spectra were used to confirm the assigned molecular weight of compounds in form of their stable fragments.

An efficient one-pot reaction procedure was employed to afford the titled compounds. A solution of 2-oxindole (0.40 g, 3.0 mmol), appropriate aromatic aldehyde (3.0 mmol) and 2-amino phenol (0.327 g, 3.0 mmol) in methanol (15 ml) with catalytic amount of conc. HCl (1.5 ml) was placed in 100-ml round-bottom flask and heated under reflux for 18 h approximately. The progress of reaction was monitored by TLC, using the solvent system ethyl acetoacetate:*n*-hexane (4:6). After completion of the reaction, the mixture was allowed to stand at room temperature overnight. The solid products so-formed were collected by filtration, dried and recrystallized with methanol. All the products thus obtained physically appeared as pure needle-shaped bright crystals, giving a single spot on the TLC plate.

#### 12-Phenyl-12,12a-dihydro-5H-benzo[2,3][1,4]oxazepino[5,6-b]indole (**1a**)

Yield: 72%; Melting range (°C): 184–188; IR (KBr; ν_max_/cm^-1^): 3078.7 (N–H str), 1706.9 (C = N str), 1328.2 (C–N str), 1232.2 (C–O–C str), 1463.2 (C = C str, aromatic), 3149.9 (C–H str, aromatic), 1613.5 (N–H bend); ^1^H NMR (CDCl_3_, 400 MHz): δ (ppm) 8.09 (s, 1H, –NH–), 7.84 (s, 1H, ArH), 7.65 (dd, 3H, ArH), 7.47 (m, 4H, ArH), 7.25 (s, 1H, ArH), 7.21 (t, 1H, ArH), 6.87 (dd, 2H, ArH), 7.04 (t, 1H, ArH), 3.49 (s, 1H, >CH–), 1.60 (s, 1H, >CH–); ^13^C NMR (CDCl_3_, 100 MHz): δ (ppm) 170.09, 159.82, 141.63, 137.80, 135.08, 132.19, 130.11, 129.89, 129.53, 128.89, 128.54, 127.64, 123.33, 122.10, 122.00, 119.50, 110.27, 109.68; MS (EI): m/z 313.2 [M+1]^+^ (15), 221.9 [M – C_6_H_4_N]^+^ (100).

#### 4-(12,12a-Dihydro-5H-benzo[2,3][1,4]oxazepino[5,6-b]indol-12-yl)phenol (**2a**)

Yield: 68%; Melting range (°C): 193–195; IR (KBr; ν_max_/cm^-1^): 3203.5 (N–H str), 1680.9 (C = N str), 1339.0 (C–N str), 1229.6 (C–O–C str), 1463.7 (C = C str, aromatic), 3203.5 (C–H str, aromatic), 1583.4 (N–H bend), 3203.5 (O–H str); ^1^H NMR (DMSO-*d*_6_, 400 MHz): δ (ppm) 10.49 (s, 1H, –NH–), 10.11 (s, 1H, ArH), 8.37 (d, 1H, ArH), 7.65 (d, 1H, ArH), 7.57 (d, 2H, ArH), 7.51 (s, 1H, ArH), 7.17 (t, 2H, ArH), 6.84 (dd, 4H, ArH), 3.42 (s, 1H, –OH), 3.37 (s, 1H, >CH–), 3.14 (s, 1H, >CH–); ^13^C NMR (DMSO-*d*_6_, 100 MHz): δ (ppm) 169.60, 159.85, 143.03, 137.14, 135.36, 132.37, 129.94, 125.53, 125.19, 122.57, 121.86, 121.54, 119.53, 116.18, 115.75, 110.47, 109.63; MS (EI): m/z 329.4 [M+1]^+^ (11), 237.9 [M – C_6_H_4_N]^+^ (100).

#### 3-(12,12a-Dihydro-5H-benzo[2,3][1,4]oxazepino[5,6-b]indol-12-yl)phenol (**3a**)

Yield: 60%; Melting range (°C):196–198; IR (KBr; ν_max_/cm^-1^): 3020.9 (N–H str), 1684.3 (C = N str), 1335.6 (C–N str), 1224.5 (C–O–C str), 1463.9 (C = C str, aromatic), 3020.9 (C–H str, aromatic), 1615.6 (N–H bend), 3170.2 (O–H str); ^1^H NMR (CDCl_3_, 400 MHz): δ (ppm) 8.27 (s, 1H, –NH–), 7.75 (s, 1H, ArH), 7.63 (d, 1H, ArH), 7.51 (d, 1H, ArH), 7.34 (t, 3H, ArH), 7.21 (d, 3H, ArH), 7.09 (s, 1H, ArH), 6.859 (m, 2H, ArH), 5.02 (s, 1H, –OH), 3.49 (s, 1H, >CH–), 2.17 (s, 1H, >CH–); ^13^C NMR (CDCl_3_, 100 MHz): δ (ppm) 172.05, 162.05, 144.05, 137.14, 135.36, 132.37, 129.94, 125.53, 125.19, 122.57, 121.86, 121.54, 119.53, 116.18, 115.75, 110.46, 109.62; MS (EI): m/z 329.4 [M+1]^+^ (16), 237.9 [M – C_6_H_4_N]^+^ (100).

#### 12-(4-Chlorophenyl)-12,12a-dihydro-5H-benzo[2,3][1,4]oxazepino[5,6-b]indole (**4a**)

Yield: 70%; Melting range (°C): 181–184; IR (KBr; ν_max_/cm^-1^): 3487.0 (N–H str), 1711.0 (C = N str), 1327.2 (C–N str), 1204.4 (C–O–C str), 1463.2 (C = C str, aromatic), 3176.1 (C–H str, aromatic), 1612.8 (N–H bend), 742.4(C–Cl str); ^1^H NMR (CDCl_3_, 400 MHz): δ (ppm) 8.79 (s, 1H, –NH–), 8.23 (d, 1H, ArH), 7.75 (s, 1H, ArH), 7.59 (dd, 3H, ArH), 7.47 (m, 3H, ArH), 7.22 (m, 1H, ArH), 7.03 (t, 1H, ArH), 6.92 (m, 2H, ArH), 3.52 (s, 1H, >CH–), 1.71 (s, 1H, >CH–); ^13^C NMR (CDCl_3_, 100 MHz): δ (ppm) 170.32, 141.98, 140.05, 136.17, 136.08, 135.78, 133.50, 132.46, 130.86, 130.40, 129.42, 129.22, 128.76, 128.31, 123.22, 122.16, 121.65, 119.60, 110.60, 109.94; MS (EI): m/z 347.9 [M+1]^+^ (19), 255.9 [M – C_6_H_4_N]^+^ (100).

#### 12-(4-Bromophenyl)-12,12a-dihydro-5H-benzo[2,3][1,4]oxazepino[5,6-b]indole (**5a**)

Yield: 80%; Melting range (°C): 166–169; IR (KBr; ν_max_/cm^-1^): 3480.8 (N–H str), 1701.4 (C = N str), 1334.1 (C–N str), 1209.2 (C–O–C str), 1460.2 (C = C str, aromatic), 3178.5 (C–H str, aromatic), 1616.5 (N–H bend), 741.2(C–Br str); ^1^H NMR (CDCl_3_, 400 MHz): δ (ppm) 8.32 (s, 1H, –NH–), 8.13 (d, 1H, ArH), 7.72 (s, 1H, ArH), 7.59 (m, 6H, ArH), 7.23 (t, 1H, ArH), 7.04 (t, 1H, ArH), 6.88 (dd, 2H, ArH), 1.63 (s, 2H, >CH–); ^13^C NMR (CDCl_3_, 100 MHz): δ (ppm) 169.99, 141.83, 136.10, 133.96, 133.62, 132.20, 131.76, 131.04, 130.43, 128.24, 124.07, 123.28, 122.20, 121.68, 110.50, 109.86; MS (EI): m/z 392.1 [M+1]^+^ (22), 301.9 [M – C_6_H_4_N]^+^ (100).

#### 12-(4-Fluorophenyl)-12,12a-dihydro-5H-benzo[2,3][1,4]oxazepino[5,6-b]indole (**6a**)

Yield: 65%; Melting range (°C): 167–170; IR (KBr; ν_max_/cm^-1^): 3143.3 (N–H str), 1706.2 (C = N str), 1328.0 (C–N str), 1221.0 (C–O–C str), 1463.0 (C = C str, aromatic), 3071.3 (C–H str, aromatic), 1613.8 (N–H bend), 1160.4 (C–F str); ^1^H NMR (CDCl_3_, 400 MHz): δ (ppm) 8.96 (s, 1H, –NH–), 8.33 (t, 1H, ArH), 7.78 (s, 1H, ArH), 7.66 (q, 2H, ArH), 7.58 (d, 1H, ArH), 7.50 (t, 1H, ArH), 7.14 (m, 4H, ArH), 6.88 (dd, 2H, ArH), 3.49 (s, 1H, >CH–), 1.77 (s, 1H, >CH–); ^13^C NMR (CDCl_3_, 100 MHz): δ (ppm) 170.56, 164.76, 162.27, 141.96, 136.45, 134.64, 131.66, 130.24, 129.17, 127.84, 123.05, 122.09, 121.75, 119.42, 116.21, 115.99, 110.62, 109.92; MS (EI): m/z 331.2 [M+1]^+^ (8), 239.9 [M – C_6_H_4_N]^+^ (100).

#### 12-(3,4,5-Trimethoxyphenyl)-12,12a-dihydro-5H-benzo[2,3][1,4]oxazepino[5,6-b]indole (**7a**)

Yield: 77%; Melting range (°C): 167–170; IR (KBr; ν_max_/cm^-1^): 3688.0 (N–H str), 1691.1 (C = N str), 1336.0 (C–N str), 1250.9 (C–O–C str), 1466.5 (C = C str, aromatic), 3169.0 (C–H str, aromatic), 2940.2 (C–H str, aliphatic), 1570.7 (N–H bend); ^1^H NMR (CDCl_3_, 400 MHz): δ (ppm) 8.12 (s, 1H, –NH–), 7.81 (dd, 2H, ArH), 7.51 (d, 1H, ArH), 7.47 (s, 1H, ArH), 7.22 (t, 1H, ArH), 7.05 (t, 1H, ArH), 6.92 (m, 3H, ArH), 3.88 (m, 9H, 3–OCH_3_), 1.61 (s, 2H, >CH–); ^13^C NMR (CDCl_3_, 100 MHz): δ (ppm) 170.16, 168.15, 153.52, 152.95, 141.71, 139.73, 138.25, 137.87, 130.27, 129.53, 128.87, 126.90, 125.84, 123.42, 122.06, 121.97, 119.28, 110.37, 109.67, 107.04, 61.28, 56.49; MS (EI): m/z 403.4 [M+1]^+^ (14), 312.0 [M – C_6_H_4_N]^+^ (100).

#### 12-(4-Methoxyphenyl)-12,12a-dihydro-5H-benzo[2,3][1,4]oxazepino[5,6-b]indole (**8a**)

Yield: 66%; Melting range (°C): 187–190; IR (KBr; ν_max_/cm^-1^): 3074.0 (N–H str), 1701.7 (C = N str), 1329.8 (C–N str), 1174.0 (C–O–C str), 1462.0 (C = C str, aromatic), 3147.3 (C–H str, aromatic), 2835.9 (C–H str, aliphatic), 1604.0 (N–H bend); ^1^H NMR (CDCl_3_, 400 MHz): δ (ppm) 9.45 (s, 1H, –NH–), 9.06 (s, 1H, ArH), 8.35 (d, 1H, ArH), 7.79 (s, 1H, ArH), 7.73 (d, 1H, ArH), 7.65 (d, 2H, ArH), 7.48 (d, 1H, ArH), 7.19 (dd, 1H, ArH), 7.00 (m, 3H, ArH), 6.88 (t, 1H, ArH), 3.86 (s, 3H, –OCH_3_), 3.51 (s, 1H, >CH–), 2.09 (s, 1H, >CH–); ^13^C NMR (CDCl_3_, 100 MHz): δ (ppm) 171.24, 168.81, 161.83, 141.83, 139.68, 137.88, 134.68, 131.73, 129.62, 128.40, 127.40, 126.05, 125.94, 124.17, 122.81, 121.85, 118.94, 114.29, 113.99, 110.55, 109.81, 55.59; MS (EI): m/z 343.3 [M+1]^+^ (17), 251.9 [M – C_6_H_4_N]^+^ (100).

#### 12-(3-Methoxyphenyl)-12,12a-dihydro-5H-benzo[2,3][1,4]oxazepino[5,6-b]indole (**9a**)

Yield: 65%; Melting range (°C): 188–190; IR (KBr; ν_max_/cm^-1^): 3148.5 (N–H str), 1714.8 (C = N str), 1464.9 (C–N str), 1274.4 (C–O–C str), 1431.1 (C = C str, aromatic), 3077.6 (C–H str, aromatic), 2947.4 (C–H str, aliphatic), 1609.4 (N–H bend); ^1^H NMR (CDCl_3_, 400 MHz): δ (ppm) 9.34 (s, 1H, –NH–), 8.31 (s, 1H, ArH), 7.81 (s, 1H, ArH), 7.65 (d, 1H, ArH), 7.53 (t, 1H, ArH), 7.38 (m, 1H, ArH), 7.18 (m, 3H, ArH), 6.98 (m, 4H, ArH), 3.91 (s, 1H, >CH–), 3.83 (s, 3H, –OCH_3_), 1.96 (s, 1H, >CH–); ^13^C NMR (CDCl_3_, 100 MHz): δ (ppm) 170.87, 168.62, 159.87, 142.08, 140.10, 137.88, 136.33, 135.29, 130.12, 129.95, 128.11, 126.85, 125.50, 123.44, 121.99, 119.46, 117.92, 115.96, 114.47, 110.59, 109.97, 55.56; MS (EI): m/z 343.2 [M+1]^+^ (14), 251.9 [M – C_6_H_4_N]^+^ (100).

#### 5-(12,12a-Dihydro-5H-benzo[2,3][1,4]oxazepino[5,6-b]indol-12-yl)-2-methoxyphenol (**10a**)

Yield: 81%; Melting range (°C): 194–197; IR (KBr; ν_max_/cm^-1^): 3170.3 (N–H str), 1686.8 (C = N str), 1466.2 (C–N str), 1204.9 (C–O–C str), 1438.4 (C = C str, aromatic), 3037.4 (C–H str, aromatic), 2893.1 (C–H str, aliphatic), 1618.9 (N–H bend), 3391.8 (O–H str); ^1^H NMR (CDCl_3_, 400 MHz): δ (ppm) 8.91 (s, 1H, –NH–), 7.79 (d, 1H, ArH), 7.76 (s, 1H, ArH), 7.60 (d, 1H, ArH), 7.48 (t, 1H, ArH), 7.28 (dd, 1H, ArH), 7.20 (t, 2H, ArH), 7.02 (m, 2H, ArH), 6.88 (m, 2H, ArH), 4.03 (s, 1H, –OH), 3.97 (s, 1H, >CH–), 3.92 (s, 3H, –OCH_3_), 3.47 (s, 1H, >CH–); ^13^C NMR (CDCl_3_, 100 MHz): δ (ppm) 170.76, 168.10, 159.87, 147.54, 146.51, 141.22, 139.77, 138.34, 129.68, 127.16, 124.32, 123.05, 121.97, 117.90, 114.93, 112.20, 110.17, 109.43, 56.31; MS (EI): m/z 359.4 [M+1]^+^ (20), 268.0 [M – C_6_H_4_N]^+^ (100).

#### 5-(12,12a-Dihydro-5H-benzo[2,3][1,4]oxazepino[5,6-b]indol-12-yl)-2-ethoxyphenol (**11a**)

Yield: 82%; Melting range (°C): 191–193; IR (KBr; ν_max_/cm^-1^): 3133.8 (N–H str), 1687.4 (C = N str), 1441.1 (C–N str), 1207.2 (C–O–C str), 1577.2 (C = C str, aromatic), 3068.7 (C–H str, aromatic), 2975.0 (C–H str, aliphatic), 1620.1 (N–H bend), 3416.2 (O–H str); ^1^H NMR (CDCl_3_, 400 MHz): δ (ppm) 8.90 (s, 1H, –NH–), 7.78 (d, 1H, ArH), 7.74 (s, 1H, ArH), 7.49 (t, 1H, ArH), 7.43 (t, 2H, ArH), 7.18 (m, 3H, ArH), 7.02 (m, 2H, ArH), 6.90 (m, 3H, ArH), 6.10 (s, 1H, >CH–), 5.96 (s, 1H, –OH), 3.49 (s, 1H, >CH–), 4.14 (s, 3H, –OC_2_H_5_); ^13^C NMR (CDCl_3_, 100 MHz): δ (ppm) 171.76, 169.10, 147.55, 146.52, 141.21, 139.77, 138.29, 129.56, 128.21, 126.98, 125.33, 124.21, 122.87, 121.97, 117.98, 114.79, 112.05, 110.19, 109.44, 56.18, 53.17; MS (EI): m/z 373.7 [M+1]^+^ (15), 282.0 [M – C_6_H_4_N]^+^ (100).

#### 12-(p-Tolyl)-12,12a-dihydro-5H-benzo[2,3][1,4]oxazepino[5,6-b]indole (**12a**)

Yield: 72%; Melting range (°C): 197–199; IR (KBr; ν_max_/cm^-1^): 3139.2 (N–H str), 1693.9 (C = N str), 1460.4 (C–N str), 1199.6 (C–O–C str), 1460.4 (C = C str, aromatic), 3070.1 (C–H str, aromatic), 2893.5 (C–H str, aliphatic), 1606.1 (N–H bend); ^1^H NMR (CDCl_3_, 400 MHz): δ (ppm) 9.38 (s, 1H, –NH–), 8.21 (d, 1H, ArH), 7.82 (s, 1H, ArH), 7.68 (d, 1H, ArH), 7.53 (t, 1H, ArH), 7.57 (d, 2H, ArH), 7.28 (t, 2H, ArH), 7.19 (t, 1H, ArH), 7.12 (t, 1H, ArH), 6.94 (d, 1H, ArH), 6.86 (t, 1H, ArH), 3.49 (s, 1H, >CH–), 2.42 (s, 3H, –CH_3_), 2.00 (s, 1H, >CH–); ^13^C NMR (CDCl_3_, 100 MHz): δ (ppm) 171.08, 141.94, 140.32, 139.96, 138.02, 132.38, 131.41, 129.86, 129.28, 128.82, 127.19, 125.67, 123.11, 122.06, 121.90, 119.25, 110.55, 109.88, 21.88; MS (EI): m/z 327.7 [M+1]^+^ (16), 235.9 [M – C_6_H_4_N]^+^ (100).

#### 12-(3-Chlorophenyl)-12,12a-dihydro-5H-benzo[2,3][1,4]oxazepino[5,6-b]indole (**13a**)

Yield: 78%; Melting range (°C): 194–197; IR (KBr; ν_max_/cm^-1^): 3184.9 (N–H str), 1708.3 (C = N str), 1463.5 (C–N str), 1201.1 (C–O–C str), 1410.3 (C = C str, aromatic), 3078.3 (C–H str, aromatic), 1613.2 (N–H bend), 783.3 (C–Cl str); ^1^H NMR (CDCl_3_, 400 MHz): δ (ppm) 9.12 (s, 1H, –NH–), 8.36 (s, 1H, ArH), 7.73 (s, 1H, ArH), 7.62 (s, 1H, ArH), 7.52 (dd, 2H, ArH), 7.45 (s, 1H, ArH), 7.41 (d, 2H, ArH), 7.25 (dd, 1H, ArH), 7.05 (t, 1H, ArH), 6.89 (d, 1H, ArH), 6.86 (t, 1H, ArH), 3.49 (s, 1H, >CH–), 1.83 (s, 1H, >CH–); ^13^C NMR (CDCl_3_, 100 MHz): δ (ppm) 170.40, 142.18, 136.89, 135.77, 134.92, 131.68, 130.57, 130.21, 129.75, 129.65, 129.04, 127.52, 123.32, 122.24, 121.49, 119.73, 110.71, 110.12; MS (EI): m/z 347.7 [M+1]^+^ (9), 255.9 [M – C_6_H_4_N]^+^ (100).

#### 12-(2-Chlorophenyl)-12,12a-dihydro-5H-benzo[2,3][1,4]oxazepino[5,6-b]indole (**14a**)

Yield: 75%; Melting range (°C): 188–190; IR (KBr; ν_max_/cm^-1^): 3185.6 (N–H str), 1713.6 (C = N str), 1462.7 (C–N str), 1230.8 (C–O–C str), 1462.7 (C = C str, aromatic), 3180.6 (C–H str, aromatic), 1614.6 (N–H bend), 747.4 (C–Cl str); ^1^H NMR (CDCl_3_, 400 MHz): δ (ppm) 9.09 (s, 1H, –NH–), 7.87 (s, 1H, ArH), 7.73 (dd, 1H, ArH), 7.50 (dd, 1H, ArH), 7.40–7.31 (m, 4H, ArH), 7.21 (t, 2H, ArH), 6.92 (d, 2H, ArH), 6.84 (t, 1H, ArH), 3.55 (s, 1H, >CH–), 1.83 (s, 1H, >CH–); ^13^C NMR (CDCl_3_, 100 MHz): δ (ppm) 170.15, 142.17, 140.44, 134.67, 134.03, 133.76, 133.16, 132.67, 131.90, 131.33, 130.96, 130.24, 129.83, 129.40, 126.83, 126.39, 123.40, 122.25, 121.58, 120.27, 110.10; MS (EI): m/z 347.9 [M+1]^+^ (18), 255.9 [M – C_6_H_4_N]^+^ (100).

#### 12-(2-Bromophenyl)-12,12a-dihydro-5H-benzo[2,3][1,4]oxazepino[5,6-b]indole (**15a**)

Yield: 77%; Melting range (°C): 187–190; IR (KBr; ν_max_/cm^-1^): 3139.5 (N–H str), 1712.6 (C = N str), 1460.7 (C–N str), 1231.6, 1201.2, (C–O–C str), 1460.7 (C = C str, aromatic), 3076.8 (C–H str, aromatic), 1614.0 (N–H bend), 741.0 (C–Br str); ^1^H NMR (CDCl_3_, 400 MHz): δ (ppm) 8.84 (s, 1H, –NH–), 7.81 (s, 1H, ArH), 7.71 (d, 3H, ArH), 7.40 (t, 1H, ArH), 7.32–7.25 (m, 3H, ArH), 7.21 (t, 1H, ArH), 6.93 (d, 2H, ArH), 6.81 (t, 1H, ArH), 3.49 (s, 1H, >CH–), 1.71 (s, 1H, >CH–); ^13^C NMR (CDCl_3_, 100 MHz): δ (ppm) 169.98, 142.06, 140.44, 136.04, 135.68, 134.03, 133.42, 132.70, 131.04, 130.46, 129.10, 127.46, 124.41, 123.44, 122.10, 121.60, 120.26, 110.60; MS (EI): m/z 392.2 [M+1]^+^ (10), 301.9 [M – C_6_H_4_N]^+^ (100).

#### 12-(2-Fluorophenyl)-12,12a-dihydro-5H-benzo[2,3][1,4]oxazepino[5,6-b]indole (**16a**)

Yield: 72%; Melting range (°C): 190–192; IR (KBr) (ν_max_/cm^-1^): 3151.8 (N–H str), 1710.8 (C = N str), 1460.3 (C–N str), 1221.8 (C–O–C str), 1460.3 (C = C str, aromatic), 3080.5 (C–H str, aromatic), 1611.9 (N–H bend), 1094.0 (C–F str); ^1^H NMR (CDCl_3_, 400 MHz): δ (ppm) 8.12 (s, 1H, –NH–), 7.90 (s, 1H, ArH), 7.71 (t, 1H, ArH), 7.45 (q, 2H, ArH), 7.25 (s, 1H, ArH), 7.22 (m, 4H, ArH), 6.87 (t, 3H, ArH), 3.49 (s, 1H, >CH–), 1.59 (s, 1H, >CH–); ^13^C NMR (CDCl_3_, 100 MHz): δ (ppm) 169.55, 159.54, 141.84, 132.67, 131.86, 130.55, 130.44, 129.99, 129.44, 124.29, 124.26, 123.55, 123.23, 122.19, 121.79, 116.49, 116.28, 110.32; MS (EI): m/z 331.5 [M+1]^+^ (14), 239.9 [M – C_6_H_4_N]^+^ (100).

### Cell culture & sulforhodamine B assay

Hep-G2 cells were grown in Roswell Park Memorial Institute media (RPMI 1640) containing 10% fetal bovine serum and 2 mM l-glutamine in T-75 flask at 37°C, 5% CO_2_, 95% air and 100% relative humidity for 24 h. After growing, 100-μl cells containing media were inoculated into 96-well plates at a concentration of 5 × 10^3^ cells/well. Separately, all the compounds to be tested were solubilized in dimethyl sulfoxide at 100 mg/ml and diluted to 1 mg/ml using water and stored frozen prior to use. Next day, 100 μl of compounds containing media was added in each well (10, 20, 40 and 80 μg/ml) and incubated at standard conditions for 48 h. To terminate the reaction, 50 μl of the cold 30% trichloroacetic acid was added and incubated at 4°C for 1 h. The supernatant was discarded; the plates were washed five-times with tap water and air dried. Furthermore, 50 μl of sulforhodamine B solution at 0.4% (w/v) in 1% acetic acid was added to each of the wells and incubated for 20 min at room temperature. After staining, the residual dye was removed by washing five-times with 1% acetic acid and the plates were air dried. The bound stain was subsequently eluted with 10 mM trizma base and the absorbance was read on a plate reader at a wavelength of 540 nm with 690-nm reference wavelength. The results were obtained in triplicate on separate plates and finally the average values were determined from these three experiments.

The percent growth inhibition was calculated using the formula [(Ti-Tz)/(C-Tz)] × 100%. The abbreviations used in formula were considered as: Time zero (Tz), control growth (C) and test growth in the presence of drug at the four concentration levels (Ti). [21,22].

### *In silico* screening

The primary structures of compounds were designed with ChemDraw Ultra 12.0 and their geometry was optimized six-times with Gauss view 5.0. On the other hand, National Centre for Biotechnology Information and Protein Data Bank were used as chemical sources to get the established five homological cancer protein targets, namely IL-2 (1Z92), IL-6 (1IL6), Caspase-3 (1QX3), Caspase-8 (1IBC) and COX-2 (4COX), respectively [23–25]. Active site was recognized with the help of CASTp database. Furthermore, *in silico* molecular docking studies of titled derivatives were performed using Autodock 4.1 along with its LGA algorithm for automated flexible ligand docking and binding energy evaluated in the form of negative kilocalorie per mole. Probable hydrogen bonds and π bonds were evaluated.

### Prediction of physiochemical properties

The Med Chem Designer and QikProp were used to predict the ADME properties of the compounds for analyzing the drug likeness of all the molecules. Chemical structure was optimized via ligprop. Furthermore, ADME profiling of all these structure was calculated. In this study, we have evaluated % ABS and Lipinski's violation [26].

### MD simulation

The dynamic simulation was used to investigate and track the behavior of used inhibitor into active site domain of IL-6. Best molecular docking pose of ligand–protein was selected for MD simulation using Elmar Krieger MD simulation tools [27]. AMBER03 force field was assigned to perform real-time MD simulation [28]. The complex was solvated with HOH model (density = 0.997 g/l) into the defined 10 A^0^ larger simulation cell boundary and the default physiological pH 7.4 were adjusted. Furthermore, we used 0.9% NaCl (physiological solution) containing Na^+^ and Cl^-^ ions concentration as a mass fraction to maintain and neutralize the simulation cell boundary. Temperature and pressure were assigned on 298 K and 1 bar, respectively. Then, the system was submitted for 3000 ps time for running the MD simulation to get snapshots (sim) trajectory. Finally, sims trajectory were analyzed and corresponding data plotted by using Sigma Plot 11.0 tools.

## Results & discussion

### *In vitro* study of anticancer activity on the Hep-G2 cell line

All the synthesized indole-fused benzooxazepines were screened against human hepatoma (Hep-G2) cancer cell lines (Table 1). Effects of the synthesized compounds (**1a–16a**) and the standard drug adriamycin (ADR) on human hepatoma cell line (Hep-G2 cells) are demonstrated in Supplementary Figure 1. The microscopic pictures (Supplementary Figure 2A–F) are showing the effect of treatments with the active compounds (**6a, 10a, 13a, 14a** and **15a**) and ADR on Hep-G2 human liver cancer cell line. Although the parent compound **1a** showed the moderate cytotoxic potential (GI_50_ = 48.3 μg/ml) against the Hep-G2 cell line; however, some of its substituted derivatives exhibited high selectivity (GI_50_ <10 μg/ml) toward the Hep-G2 cell line. Activity results proved that substitutions at 2, 3 and 4 positions of the phenyl ring play a crucial role in imparting the anticancer activity. The C-4 substitutions (–OH, –Cl, –Br, –OCH_3_ and –CH_3_) on phenyl ring led to compounds **2a, 4a, 5a, 8a** and **12a** without any significant improvement in cytotoxicity, except the compounds **6a**, possessing more electronegative group (F), exhibited better cytotoxicity (GI_50_ <10 μg/ml). Similarly, the C-3 substitutions with Cl on phenyl ring led to compound **13a** with better cytotoxicity profile (GI_50_ <10 μg/ml). In addition, the C-2 substitutions with Cl and Br on phenyl ring led to compounds **14a** and **15a** with significant improvement in cytotoxicity (GI_50_ < 10 μg/ml), whereas the C-2 substitutions with more electronegative group (F) led to compound **16a** with slightly reduced cytotoxicity profile (GI_50_ = 10.7). In general, it may be concluded that the halogenations of phenyl ring were more beneficial for the anticancer activity when compared with the parent compound **1a**. In conjugation with this, the introduction of methoxy group at C-3 and C-4 position led to compounds **8a** and **9a** with slightly improved activity (GI_50_ = 15.8 and 36.7), whereas 3,4,5-trimethoxy substitution led to compound **7a** with slightly decreased anticancer activity (GI_50_ = 52.6). The hydroxylation or methylation of phenyl ring (compounds: **2a**, **3a** and **12a**) is detrimental for the cytotoxic activity. However, while retaining the important methoxy substitution at C-4 position, hydroxylation at C-3 position led to compound **10a** with an appreciable improvement in cytotoxic potential (GI_50_ <10 μg/ml), whereas alteration of methoxy group with ethoxy group (compound **11a**) again lost the cytotoxic potential. Interestingly, the growth curve of *in vitro* data suggested that, at 10 μg/ml concentrations of active compounds, the % control growths are 50% or below 50%, but they do not fall in the negative value of % control growth. Thus, for the future, it might be expected that all the active compounds of the series will kill the cancerous cell while minimizing the normal cell death.

### *In silico* study of anticancer activity

*In silico* molecular docking was performed using five established liver cancer targets, namely IL-2, IL6, COX-2, Caspase-3 and Caspase-8 via Autodoc 4.1 along with LGA algorithm parameter for automated flexible ligand docking. Docking images for the active compounds **6a, 10a, 13a, 14a** and **15a** with the related targets IL-2, IL-6, COX-2, Caspase-3 and Caspase-8 are illustrated in Supplementary Figure 3 that indicate the amino acids interaction with the ligands, H- and π-bonds and their bond lengths. The binding affinity (kcal/mol), number of H- and π-bonds, and amino acids interaction for only active compounds are shown in Table 2, whereas the binding affinity and (kcal/mol) and amino acids interactions for all the synthesized compounds are shown in Supplementary Table 1. The molecular docking studies of all the compounds had shown the good binding affinity with the selected targets. Predominantly, compounds **6a, 10a, 13a, 14a** and **15a** exhibited potent affinity with selected molecular targets having interaction energies ranges from -6.6 to -10.9 kcal/mol with various molecular targets. Compound **6a** displayed the good binding affinity with the COX-2 (-10.5 kcal/mol and 15 π-bonds), IL-2 (-8.7 kcal/mol, 2H and five π-bonds), IL-6 (-8.3 kcal/mol and nine π-bonds), Caspase-3 (-7.1 kcal/mol, 1H and four π-bonds) and Caspase-8 (-7.0 kcal/mol, 1H and seven π-bonds). A similar fashion was observed for the compounds **13a** and **14a**; however, compound **10a** manifested somewhat less affinity toward Caspase-3 (-6.8 kcal/mol and four π-bonds) and Caspase-8 (-6.7 kcal/mol, 2H and eight π-bonds), whereas compound **15a** exhibited less affinity toward Caspase-3 (-6.6 kcal/mol, 1H and eight π-bonds). Although all of the active synthesized compounds have moderate-to-excellent binding affinities toward IL-2, IL6, COX-2, Caspase-3 and Caspase-8, the binding energies on COX-2 receptor site are predominantly high (9.1–10.9 kcal/mol). From this, it might be predicted that the promising cytotoxic potential of these active compounds, which was confirmed by the *in vitro* anticancer activity on human HCC Hep-G2 cell line, might be better mediated through COX-2-dependent mechanism (Tables 1 & 2).

### Prediction of ADME properties

A computational study was performed via QikProp tools to predict the physiochemical properties of the compounds **1a**–**16a**. The ranges of the calculated property of the molecules with average value are shown in Table 3. Herein, we also predicted the percentage of absorption (% ABS), rotatable bonds (n-ROTB), number of hydrogen bond donors (n-OHNH), number of hydrogen bond acceptors (n-OH), predicted octanol/water partition coefficient (QPlogPo/w) and Lipinski's violation. It was investigated that the synthesized compounds showed the % ABS ranging from 85 to 100%. Moreover, all of the synthesized compounds followed the violated Lipinski's parameters. Other parameter such as QPlogPo/w predicts octanol/water partition coefficient, which was found within the accepted range of -2.0 to 6.5.

### MD simulation

MD simulation of compound **14a** was performed with IL-6. This ligand displayed the good binding affinity, H-bond and contraction with back bone structure of IL-6. So, we decided to study the influences of compound **14a** into the active site domain of IL-6 on the structure protein. Root-mean-square deviation (RMSD), potential energy and binding energy of the IL-6 with compound **14a** containing complex were calculated by MD trajectory frames. The RMSD, potential energy and binding energy are profiled in Figure 5. Through the graphic profile, we observed the structural stability of backbone structure throughout the MD simulation. No more fluctuation was observed into the RMSD after the time (100 ps), which indicates the stability of back bone structure with ligand near the 1000 ps time in MD simulation. Potential energy and binding energy of complex were calculated with time, which indicated that the potential energy (kJ/mol) do not show more fluctuation after 100 ps time, whereas average complex binding energy was observed near -1.6 kg/mol. The fluctuation into the residue of back bone structure is shown in Figure 4.

Finally, we performed these calculations of data, where we found the structural stability of compound **14a** along with IL-6 into active site domain.

## Conclusion

We have synthesized a series of novel indole-fused benzooxazepines that displayed a potent cytotoxicity against the Hep-G2 cell line for the treatment of HCC. While considering all the newly synthesized compounds together, it may be concluded that the fusion of indole-fused benzooxazapines with substituted phenyl ring as a hydrophobic side chain establishes an important pharmacophoric structure and the positions 2, 3 and 4 of the phenyl side chain are the key reactive sites that could be altered with different groups to elicit valuable anticancer profiles. More precisely, the substitutions with more electronegative halogen atoms at phenyl ring directly attached to the indole-fused benzooxazepine led to compounds **6a** and **13a–15a**, eliciting enhanced cytotoxic potential with GI_50_ < 10 μg/ml, which was also supported by molecular docking study. In addition, 3-hydroxy-4-methoxy phenyl-substituted indole-used benzooxazepine led to compound **10a**, which also exhibited enhanced cytotoxic potential with GI_50_ < 10 μg/ml. Computation study demonstrated good oral absorption and human albumin protein binding. Hence, these titled compounds might be stable in the pharmaceutical dosage form. Moreover, the titled compounds contain a novel pharmacophore incorporating indole-fused benzooxazepine that have never been synthesized prior to this study to our knowledge; so, the present scaffold may emerge as an anticancer lead for the future.

The *in vivo* anticancer studies of potent compounds in the series, studies to improve anticancer activity and toxicity profiling of indole-fused benzooxazepines are in progress.

## Future perspective

Cancer is still a big challenge for researchers and there is an immense need for exploration and development of novel lead compounds. Ergo, there is a desideratum for more potent, less toxic and less expensive anticancer drugs. To accomplish this goal, indole, azepine and six-membered flexible rings are getting much attention for cancer therapy. The synthesized indole-fused benzooxazepines attached with six-membered flexible ring might be counted as primary lead molecules for future modification and optimization, to afford potential anticancer drugs. Interestingly, from the growth curve of *in vitro* data, it might be expected in the future that all the active compounds of series will kill the cancerous cell while minimizing the normal cell death. In addition, a feasible one-pot-efficient synthetic approach for the proposed derivatives will make it cost effective. Lastly, these newly synthesized lead compounds need to go through further *in vivo* anticancer activity and toxicity profiling for better clarification of suitability of titled compounds for the treatment of various types of cancer.

**Table 1.** ***In vitro* cytotoxicity data of synthesized compounds against human hepatoma (Hep-G2) cancer cell lines.**

**Compound code**	**R_1_**	**R_2_**	**R_3_**	**R_4_**	**GI_50_ (μg/ml)**	**LC_50_ (μg/ml)**	**TGI (μg/ml)**
1a	–H	–H	–H	–H	48.3	>80	>80
2a	–H	–H	–OH	–H	NE	NE	NE
3a	–H	–OH	–H	–H	>80	>80	>80
4a	–H	–H	–Cl	–H	>80	>80	>80
5a	–H	–H	–Br	–H	>80	>80	>80
6a	–H	–H	–F	–H	<10	NE	NE
7a	–H	–OCH_3_	–OCH_3_	–OCH_3_	52.6	>80	>80
8a	–H	–H	–OCH_3_	–H	36.7	>80	>80
9a	–H	–OCH_3_	–H	–H	15.8	>80	>80
10a	–H	–OH	–OCH_3_	–H	<10	>80	>80
11a	–H	–OH	–OC_2_H_5_	–H	NE	NE	NE
12a	–H	–H	–CH_3_	–H	57.0	>80	>80
13a	–H	–Cl	–H	–H	<10	>80	>80
14a	–Cl	–H	–H	–H	<10	>80	>80
15a	–Br	–H	–H	–H	<10	NE	39.9
16a	–F	–H	–H	–H	10.7	NE	NE
ADR					<10	NE	<10

GI_50_ value of ≤10 μg/ml (or 1 μmolar) is considered to demonstrate activity in case of pure compounds (synthetic compound).

‘NE’ stands for ‘Not Effective’ even at the concentration >80 μg/ml.

GI_50_ = Concentration of drug causing 50% inhibition of cell growth.

LC_50_ = Concentration of drug causing 50% cell kill.

TGI = Concentration of drug causing total inhibition of cell growth.

ADR = Adriamycin, positive control compound.

**Table 2.** **Docking affinity of active compounds with assigned anticancer receptors.**

**Ligands**	**Receptors**	**Binding affinity (kcal/mol)**	**Amino acids involved in interaction**	**H-bonds**	**π-bonds**
6a	IL-2	-8.7	ARG A 38 THR A 41 ASP B 6 PRO B 7 PHE B 15 LYS B 16 THR B 115 GLU B 116 ARG B 117 ILE B 118 TYR B 119 PHE B 121	2	5
	IL-6	-8.3	ASN A 62 LEU A 63 ASN A 64 LEU A 65 PRO A 66 LYS A 67 MET A 68 LEU A 166 ARG A 169 SER A 170 GLU A 173 PHE A 174	0	9
	COX-2	-10.5	ASN D 34 CYS D 36 CYS D 37 ASN D 39 PRO D 40 CYS D 41 GLU D 46 CYS D 47 TYR D 130 GLY D 135 TYR D 136 LYS D 137 LEU D 152 PRO D 153 PRO D 154 VAL D 155 ALA D 156 GLN D 461 GLU D 465	0	15
	Caspase-3	-7.1	THR A 62 SER A 63 ARG A 64 SER A 65 HIS A 121 CYS A 163 LEU A 168 TYR A 204 TRP A 206 ARG A 207 SER A 209 PHE A 256 HOH A 645 HOH A 648 HOH A 708 HOH A 733 HOH A 736	1	4
	Caspase-8	-7.0	LYS A 158 ARG A 161 THR A 162 ARG A 163 GLN A 194 ASN A 195 LEU A 196 GLY A 197 TYR A 198 SER A 199 VAL A 200 HOH A 601 HOH A 636	1	3
10a	IL-2	-7.5	LYS A 43 TYR A 45 ASP A 109 GLU A 110 THR A 111 GLU B 29 CYS B 30 LYS B 31 ARG B 32 GLY B 33 PHE B 34 ARG B 35	3	4
	IL-6	-7.5	GLU A 43 THR A 44 LYS A 47 SER A 48 LEU A 102 ARG A 105 PHE A 106 GLU A 107 SER A 108 GLN A 157 ASP A 161 THR A 164	1	6
	COX-2	-10.9	TRP C 323 GLN C 327 ASN D 34 CYS D 36 CYS D 37 ASN D 39 CYS D 41 GLU D 46 CYS D 47 MET D 48 SER D 49 TYR D 130 GLY D 135 TYR D 136 PRO D 153 VAL D 155 ALA D 156 CYS D 159 GLN D 461	2	12
	Caspase-3	-6.8	GLU A 43 ARG A 75 ARG A 79 LYS A 82 TYR A 83 GLU A 84 VAL A 85 HOH A 617 HOH A 667 HOH A 685 HOH A 716 HOH A 722 HOH A 741	0	4
	Caspase-8	-6.7	GLY A 291 VAL A 292 VAL A 293 THR B 334 ASN B 337 PHE B 377 GLU B 378 PRO B 380 MET B 386 HOH B 623	2	8
13a	IL-2	-8.5	ARG A 38 ASP B 6 PRO B 7 PHE B 15 THR B 115 GLU B 116 ARG B 117 ILE B 118 TYR B 119 PHE B 121	1	5
	IL-6	-7.6	GLU A 43 THR A 44 LYS A 47 SER A 48 LEU A 102 ARG A 105 PHE A 106 GLU A 107 SER A 108 ASP A 161 THR A 164	1	4
	COX-2	-10.6	ASN B 34 CYS B 36 CYS B 37 ASN B 39 PRO B 40 CYS B 41 GLU B 46 CYS B 47 TYR B 130 GLY B 135 TYR B 136 LYS B 137 LEU B 152 PRO B 153 PRO B 154 VAL B 155 ALA B 156 GLN B 461	2	12
	Caspase-3	-7.3	SER A 65 TYR A 204 TRP A 206 ARG A 207 ASN A 208 SER A 209 TRP A 214 SER A 249 PHE A 250 SER A 251 ASP A 253 PHE A 256 HOH A 645 HOH A 665 HOH A 684 HOH A 696 HOH A 736	0	6
	Caspase-8	-7.8	ALA A 141 TRP A 145 PRO A 154 ILE A 155 MET A 156 ASP A 157 PHE B 401 HIS B 404	0	7
14a	IL-2	-8.7	ARG A 38 ASP B 6 PRO B 7 PHE B 15 LYS B 16 THR B 115 GLU B 116 ARG B 117 ILE B 118 TYR B 119 PHE B 121	2	7
	IL-6	-8.4	ASN A 62 LEU A 63 ASN A 64 LEU A 65 PRO A 66 LYS A 67 MET A 68 LEU A 166 ARG A 169 SER A 170 GLU A 173 PHE A 174	0	10
	COX-2	-10.6	CYS D 36 CYS D 37 SER D 38 ASN D 39 PRO D 40 CYS D 41 GLY D 45 GLU D 46 CYS D 47 MET D 48 TYR D 130 GLY D 135 TYR D 136 LYS D 137 PRO D 153 PRO D 154 VAL D 155 ALA D 156 GLN D 461	1	10
	Caspase-3	-7.4	THR A 62 SER A 63 ARG A 64 SER A 65 TYR A 204 TRP A 206 ARG A 207 SER A 209 SER A 249 PHE A 250 SER A 251 PHE A 256 HOH A 645 HOH A 665 HOH A 684 HOH A 736	0	6
	Caspase-8	-8.2	LEU A 138 ALA A 141 GLN A 142 TRP A 145 ILE A 155 MET A 156 ASP A 157 PHE B 401 HIS B 404	1	12
15a	IL-2	-7.1	ARG A 38 ASP B 6 PRO B 7 GLU B 9 ILE B 10 ALA B 13 THR B 14 PHE B 15 GLU B 116 ARG B 117 ILE B 118 TYR B 119	0	5
	IL-6	-7.4	GLU A 43 THR A 44 LYS A 47 SER A 48 LEU A 102 ARG A 105 PHE A 106 GLU A 107 SER A 108 ASP A 161 THR A 164	1	5
	COX-2	-9.1	PRO C 127 PRO C 128 PHE C 142 LEU C 145 GLN C 374 ASN C 375 ARG C 376 LEU D 145 GLY D 225 HIS D 226 GLY D 227 TYR D 373 GLN D 374 ASN D 375 ARG D 376 GLY D 536 ASN D 537 PRO D 538	2	3
	Caspase-3	-6.6	ARG A 75 ARG A 79 LYS A 82 TYR A 83 GLU A 84 VAL A 85 HOH A 617 HOH A 685 HOH A 716 HOH A 722	1	8
	Caspase-8	-8.2	LEU A 138 ALA A 141 GLN A 142 TRP A 145 ILE A 152 PRO A 154 ILE A 155 MET A 156 ASP A 157 LYS A 158 PHE B 401 HIS B 404	1	11

**Table 3.** **Pharmacokinetic parameters important for oral bioavailability and protein-binding parameters of synthesized compounds.**

**Comp.**	**% ABS**	**n-ROTB**	**MW**	**Volume**	**n-OHNH donors**	**n-OH acceptors**	**Lipinski's violation**	**QPlogPo/w**
Rule	>80% is high <25% is poor	0–15	<500	500.0 to 2000.0	<5	<10	≤1	-2.0 to 6.5
1a	100	1	328.4	1001.3	2	2.5	0	4.1
2a	100	1	328.4	998.1	2	2.5	0	4.2
3a	100	0	348.8	1044.8	2	2.8	0	4.5
4a	100	0	391.3	1028.2	1	1.8	1	5.5
5a	100	0	330.4	994.8	1	1.8	1	5.2
6a	95	3	404.5	1216.2	2	5.0	0	4.4
7a	100	1	342.4	1054.3	1	2.5	1	5.1
8a	100	1	342.4	1050.1	1	2.5	1	5.1
9a	100	2	358.4	1071.6	2	3.3	0	4.4
10a	100	3	372.4	1146.7	2	3.3	0	4.8
11a	100	0	326.4	1038.4	1	1.8	1	5.3
12a	100	0	346.8	1018.9	1	1.8	1	5.4
12a	100	0	346.8	1011.4	1	1.8	1	5.3
13a	100	0	391.3	1007.3	1	1.8	1	5.3
14a	85	0	330.4	989.1	1	1.8	1	5.1
15a	100	1	328.4	1001.3	2	2.5	0	4.1
16a	100	1	328.4	1001.3	2	2.5	0	4.1

% ABS: Percentage of absorption; MW: Molecular weight of the molecule; n-OHNH donors: Number of hydrogen bond donors; n-OH: Number of hydrogen bond acceptors; n-ROTB: Rotatable bonds; QPlogPo/w: Predicted octanol/water partition coefficient; Volume: Total solvent-accessible volume in cubic angstroms using a probe with a 1.4 Å radius.

**Executive summary**

Indole and seven-membered azepine rings have been individually reported for their potential benefits in the prevention of different cancers.
Indole-fused benzooxazepines incorporated with a flexible substituted phenyl ring were synthesized by one-pot three-component approach as new pharmacophoric lead compounds for the treatment of hepatocellular carcinoma.
*In vitro* study on the Hep-G2 cell line showed that substitutions with more electronegative groups at phenyl ring directly attached with indole-fused benzooxazepine generally elicited enhanced cytotoxic potential.
Molecular docking on related targets including IL-2, IL-6, COX-2, Caspase-3 and Caspase-8 receptor site supported the *in vitro* study on the Hep-G2 cell line; ADMET profiling showed the better oral absorption; and MD simulation study showed the good structural stability of compound **14a** along with IL-6 into active site domain.
