# Supplementary material for: Indole-fused benzooxazepines: a new structural class of anticancer agents
Source: Future Sci OA. 2017 Jan 4;3(1):FSO168. doi: 10.4155/fsoa-2016-0079 (PMC5351710; doi:10.4155/fsoa-2016-0079)
Supplement: Supplementary file 1 [file fsoa-03-168-s1.doc]

**Supplementary Data**

**Figure S1:** Effect of synthesized compounds (**1a-16a**) on Human hepatoma cell line (Hep-G2 cells)


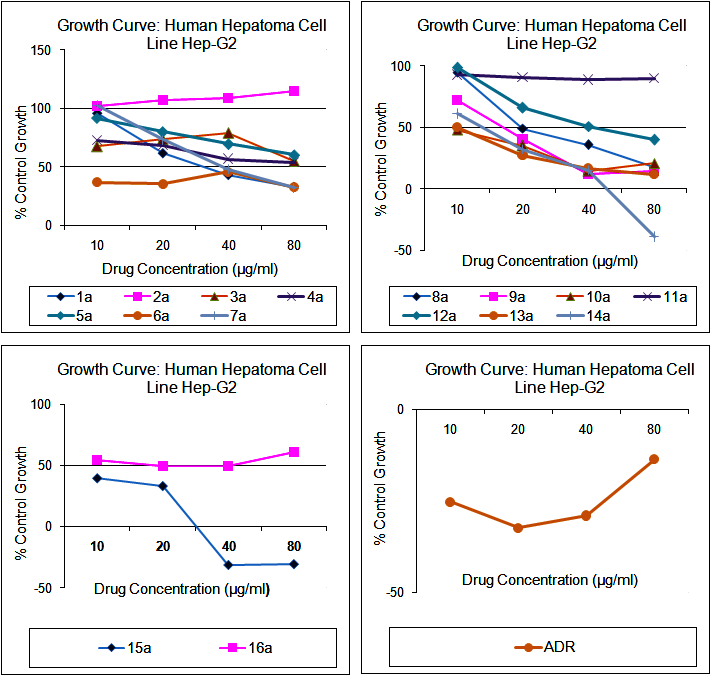


**Figure S2.** Microscopic pictures of Hep-G2 Human liver cancer cell line with various treatment of drugs: **A to E** = Compounds 6a, 10a, 13a, 14a, and 15a, respectively and **P** = positive control (Adriamycin).


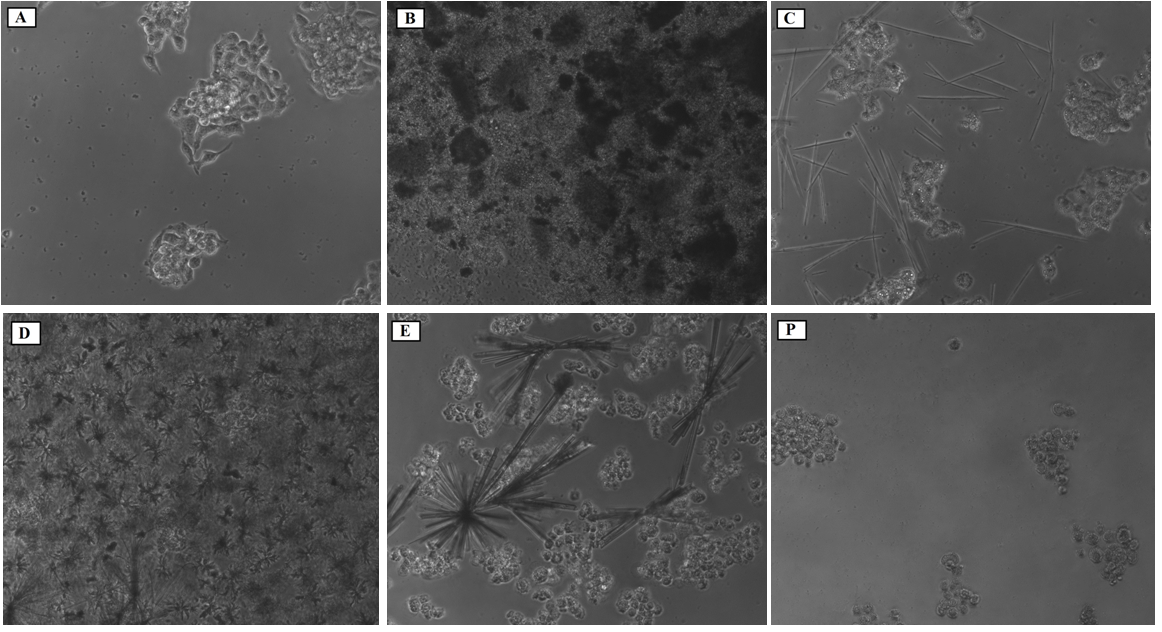


**Figure S3.** Docking poses of active compounds along with contracted amino acids, bonds and distance.

**
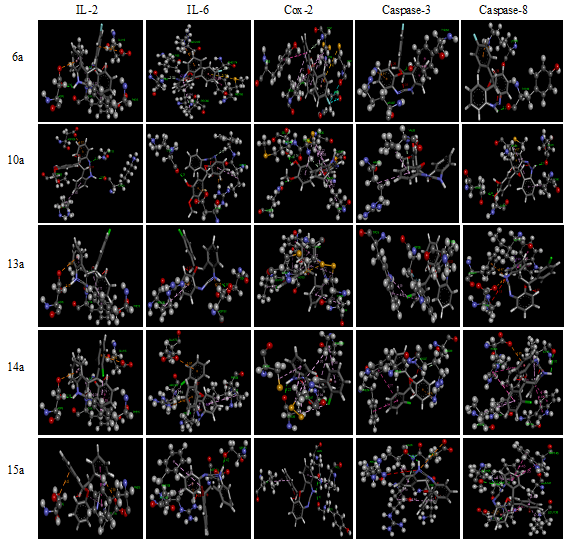
**

**Table S1. Molecular docking of designed ligands with corresponding anticancer targets**

| **Ligands** | **Receptors** | **Binding affinity [kcal/**  **mol]** | **Amino acids involved in interaction** |
| --- | --- | --- | --- |
| **1a** | IL-2 | -8.4 | ARG A 38 THR A 41 ASP B 6 PRO B 7 PHE B 15 LYS B 16 GLU B 116 ARG B 117 ILE B 118 TYR B 119 PHE B 121 |
|  | IL-6 | -7.5 | GLU A 43 THR A 44 LYS A 47 SER A 48 LEU A 102 ARG A 105 PHE A 106 GLU A 107 SER A 108 ASP A 161 THR A 164 |
|  | COX-2 | -10.3 | ASN D 34 CYS D 36 CYS D 37 ASN D 39 PRO D 40 CYS D 41 GLU D 46 CYS D 47 TYR D 130 GLY D 135 TYR D 136 LYS D 137 LEU D 152 PRO D 153 PRO D 154 VAL D 155 ALA D 156 GLN D 461 |
|  | Caspase-3 | -6.8 | THR A 62 SER A 63 SER A 65 TYR A 204 TRP A 206 ARG A 207 SER A 209 SER A 249 PHE A 250 SER A 251 PHE A 256 HOH A 645 HOH A 665 HOH A 684 HOH A 696 HOH A 733 HOH A 736 |
|  | Caspase-8 | -7.1 | LEU A 138 ALA A 141 TRP A 145 ILE A 152 PRO A 154 ILE A 155 MET A 156 ASP A 157 PHE B 401 HIS B 404 |
| **2a** | IL-2 | -8.5 | ARG A 38 THR A 41 ASP B 6 PRO B 7 PHE B 15 LYS B 16 THR B 115 GLU B 116 ARG B 117 ILE B 118 TYR B 119 PHE B 121 |
|  | IL-6 | -7.9 | ASN A 62 LEU A 63 ASN A 64 LEU A 65 PRO A 66 LYS A 67 MET A 68 LEU A 166 ARG A 169 SER A 170 GLU A 173 PHE A 174 |
|  | COX-2 | -10.7 | CYS C 36 ASN C 39 PRO C 40 CYS C 41 GLN C 42 ASN C 43 ARG C 44 GLY C 45 GLU C 46 CYS C 47 TYR C 130 LEU C 152 PRO C 153 GLN C 461 GLU C 465 LYS C 468 ARG C 469 |
|  | Caspase-3 | -7.3 | SER A 65 TYR A 204 TRP A 206 ARG A 207 ASN A 208 SER A 209 TRP A 214 SER A 249 PHE A 250 SER A 251 PHE A 256 HOH A 645 HOH A 684 HOH A 696 |
|  | Caspase-8 | -6.9 | GLY A 131 LYS A 158 ARG A 161 THR A 162 ARG A 163 GLN A 194 ASN A 195 LEU A 196 GLY A 197 TYR A 198 SER A 199 VAL A 200 HOH A 601 HOH A 636 |
| **3a** | IL-2 | -8.4 | ARG A 38 ASP B 6 PRO B 7 GLU B 9 ALA B 13 THR B 14 PHE B 15 LYS B 16 GLU B 116 ARG B 117 ILE B 118 TYR B 119 PHE B 121 |
|  | IL-6 | -8.1 | ASN A 62 LEU A 63 ASN A 64 LEU A 65 PRO A 66 LYS A 67 MET A 68 LEU A 166 ARG A 169 SER A 170 GLU A 173 PHE A 174 |
|  | COX-2 | -10.7 | ASN A 34 CYS A 36 CYS A 37 ASN A 39 PRO A 40 CYS A 41 GLU A 46 CYS A 47 MET A 48 SER A 49 TYR A 130 GLY A 135 TYR A 136 PRO A 153 VAL A 155 ALA A 156 CYS A 159 GLN A 461 TRP B 323 GLN B 327 |
|  | Caspase-3 | -7.2 | THR A 62 SER A 63 ARG A 64 SER A 65 HIS A 121 CYS A 163 LEU A 168 TYR A 204 TRP A 206 ARG A 207 SER A 209 PHE A 256 HOH A 645 HOH A 648 HOH A 708 HOH A 711 HOH A 733 HOH A 736 |
|  | Caspase-8 | -6.8 | LEU A 138 GLN A 142 TRP A 145 ILE A 152 TYR A 153 PRO A 154 ILE A 155 MET A 156 PHE B 401 HIS B 404 |
| **4a** | IL-2 | -8.7 | ARG A 38 THR A 41 ASP B 6 PRO B 7 PHE B 15 LYS B 16 THR B 115 GLU B 116 ARG B 117 ILE B 118 TYR B 119 PHE B 121 |
|  | IL-6 | -7.8 | ASN A 62 LEU A 63 ASN A 64 LEU A 65 PRO A 66 LYS A 67 MET A 68 LEU A 166 ARG A 169 SER A 170 GLU A 173 PHE A 174 SER A 177 |
|  | COX-2 | -11.0 | ASN D 34 CYS D 36 CYS D 37 ASN D 39 PRO D 40 CYS D 41 GLN D 42 GLU D 46 CYS D 47 TYR D 130 GLY D 135 TYR D 136 LYS D 137 LEU D 152 PRO D 153 PRO D 154 VAL D 155 ALA D 156 GLN D 461 GLU D 465 |
|  | Caspase-3 | -6.4 | GLY A 171 ILE A 172 GLU A 173 ALA A 244 THR A 245 LYS A 260 GLN A 261 ILE A 26???H A 602 |
|  | Caspase-8 | -7.8 | LEU A 138 TRP A 145 PRO A 154 ILE A 155 MET A 156 ASP A 157 PHE B 401 HIS B 404 |
| **5a** | IL-2 | -7.2 | GLU A 68 ASN A 71 LEU A 72 ASP B -1 PRO B 0 LEU B 2 GLY B 23 MET B 25 TYR B 43 LEU B 45 CYS B 46 THR B 47 |
|  | IL-6 | -7.6 | ASN A 62 LEU A 63 LEU A 65 PRO A 66 LYS A 67 MET A 68 ALA A 69 LEU A 166 SER A 170 GLU A 173 PHE A 174 |
|  | COX-2 | -11.1 | ASN A 34 CYS A 36 CYS A 37 ASN A 39 PRO A 40 CYS A 41 GLU A 46 CYS A 47 TYR A 130 GLY A 135 TYR A 136 LYS A 137 LEU A 152 PRO A 153 PRO A 154 VAL A 155 ALA A 156 GLN A 461 GLU A 465 |
|  | Caspase-3 | -6.4 | GLY A 171 ILE A 172 GLU A 173 ALA A 244 THR A 245 LYS A 260 GLN A 261 ILE A 262 HOH A 602 |
|  | Caspase-8 | -7.3 | LEU A 138 ALA A 141 GLN A 142 TRP A 145 ILE A 155 MET A 156 ASP A 157 PHE B 401 HIS B 404 |
| **6a** | IL-2 | -8.7 | ARG A 38 THR A 41 ASP B 6 PRO B 7 PHE B 15 LYS B 16 THR B 115 GLU B 116 ARG B 117 ILE B 118 TYR B 119 PHE B 121 |
|  | IL-6 | -8.3 | ASN A 62 LEU A 63 ASN A 64 LEU A 65 PRO A 66 LYS A 67 MET A 68 LEU A 166 ARG A 169 SER A 170 GLU A 173 PHE A 174 |
|  | COX-2 | -10.5 | ASN D 34 CYS D 36 CYS D 37 ASN D 39 PRO D 40 CYS D 41 GLU D 46 CYS D 47 TYR D 130 GLY D 135 TYR D 136 LYS D 137 LEU D 152 PRO D 153 PRO D 154 VAL D 155 ALA D 156 GLN D 461 GLU D 465 |
|  | Caspase-3 | -7.1 | THR A 62 SER A 63 ARG A 64 SER A 65 HIS A 121 CYS A 163 LEU A 168 TYR A 204 TRP A 206 ARG A 207 SER A 209 PHE A 256 HOH A 645 HOH A 648 HOH A 708 HOH A 733 HOH A 736 |
|  | Caspase-8 | -7.0 | LYS A 158 ARG A 161 THR A 162 ARG A 163 GLN A 194 ASN A 195 LEU A 196 GLY A 197 TYR A 198 SER A 199 VAL A 200 HOH A 601 HOH A 636 |
| **7a** | IL-2 | -7.0 | LYS A 43 TYR A 45 ASP A 109 GLU A 110 THR A 111 GLU B 29 CYS B 30 LYS B 31 ARG B 32 GLY B 33 PHE B 34 ARG B 35 ARG B 36 GLU B 113 ALA B 114 THR B 115 |
|  | IL-6 | -6.9 | GLU A 43 THR A 44 LYS A 47 SER A 48 LEU A 102 ARG A 105 PHE A 106 GLU A 107 SER A 108 ASP A 161 THR A 164 |
|  | COX-2 | -9.8 | LEU A 366 PHE A 367 GLN A 369 GLN A 370 PHE A 371 TYR A 373 PRO A 542 GLN A 543 THR B 118 SER B 121 TYR B 122 ILE B 124 ASP B 125 SER B 126 GLN B 369 GLN B 370 PHE B 371 GLN B 372 LYS B 532 |
|  | Caspase-3 | -7.2 | SER A 65 TYR A 204 TRP A 206 ARG A 207 ASN A 208 SER A 209 TRP A 214 SER A 249 PHE A 250 SER A 251 ASP A 253 PHE A 256 HOH A 645 HOH A 665 HOH A 684 HOH A 696 HOH A 736 |
|  | Caspase-8 | -7.3 | LEU A 138 ALA A 141 GLN A 142 TRP A 145 ILE A 155 MET A 156 ASP A 157 LYS A 158 SER A 159 ARG A 161 PHE B 401 HIS B 404 |
| **8a** | IL-2 | -8.4 | ARG A 38 THR A 41 ASP B 6 PRO B 7 PHE B 15 LYS B 16 THR B 115 GLU B 116 ARG B 117 ILE B 118 TYR B 119 PHE B 121 |
|  | IL-6 | -7.4 | ALA A 59 GLU A 60 ASN A 62 LEU A 63 ASN A 64 LEU A 65 PRO A 66 MET A 68 LEU A 166 ARG A 169 SER A 170 GLU A 173 PHE A 174 |
|  | COX-2 | -10.4 | ASN A 39 PRO A 40 CYS A 41 GLN A 42 ARG A 44 GLY A 45 GLU A 46 CYS A 47 TYR A 130 LYS A 137 ALA A 151 LEU A 152 PRO A 153 GLN A 461 GLU A 465 LYS A 468 ARG A 469 LYS B 546 |
|  | Caspase-3 | -6.5 | TRP A 206 ARG A 207 ASN A 208 TRP A 214 GLU A 248 SER A 249 PHE A 250 SER A 251 PHE A 252 HOH A 645 HOH A 684 HOH A 696 HOH A 719 |
|  | Caspase-8 | -7.0 | LEU A 138 ALA A 141 GLN A 142 TRP A 145 ILE A 152 PRO A 154 ILE A 155 MET A 156 ASP A 157 PHE B 401 HIS B 404 |
| **9a** | IL-2 | -8.5 | ARG A 38 ASP B 6 PRO B 7 GLU B 9 ILE B 10 ALA B 13 THR B 14 PHE B 15 LYS B 16 THR B 115 GLU B 116 ARG B 117 ILE B 118 TYR B 119 PHE B 121 |
|  | IL-6 | -7.5 | ALA A 59 GLU A 60 ASN A 62 LEU A 63 ASN A 64 LEU A 65 PRO A 66 MET A 68 LEU A 166 ARG A 169 SER A 170 GLU A 173 PHE A 174 |
|  | COX-2 | -10.7 | CYS A 36 CYS A 37 ASN A 39 PRO A 40 CYS A 41 GLN A 42 ARG A 44 GLY A 45 GLU A 46 CYS A 47 TYR A 130 LEU A 152 PRO A 153 GLN A 461 GLU A 465 LYS A 468 ARG A 469 |
|  | Caspase-3 | -7.0 | SER A 65 TYR A 204 TRP A 206 ARG A 207 ASN A 208 SER A 209 TRP A 214 SER A 249 PHE A 250 SER A 251 PHE A 256 HOH A 645 HOH A 684 HOH A 696 HOH A 736 |
|  | Caspase-8 | -6.9 | LEU A 138 ALA A 141 GLN A 142 TRP A 145 ILE A 155 MET A 156 ASP A 157 LYS A 158 PHE B 401 HIS B 404 |
| **10a** | IL-2 | -7.5 | LYS A 43 TYR A 45 ASP A 109 GLU A 110 THR A 111 GLU B 29 CYS B 30 LYS B 31 ARG B 32 GLY B 33 PHE B 34 ARG B 35 |
|  | IL-6 | -7.5 | GLU A 43 THR A 44 LYS A 47 SER A 48 LEU A 102 ARG A 105 PHE A 106 GLU A 107 SER A 108 GLN A 157 ASP A 161 THR A 164 |
|  | COX-2 | -10.9 | TRP C 323 GLN C 327 ASN D 34 CYS D 36 CYS D 37 ASN D 39 CYS D 41 GLU D 46 CYS D 47 MET D 48 SER D 49 TYR D 130 GLY D 135 TYR D 136 PRO D 153 VAL D 155 ALA D 156 CYS D 159 GLN D 461 |
|  | Caspase-3 | -6.8 | GLU A 43 ARG A 75 ARG A 79 LYS A 82 TYR A 83 GLU A 84 VAL A 85 HOH A 617 HOH A 667 HOH A 685 HOH A 716 HOH A 722 HOH A 741 |
|  | Caspase-8 | -6.7 | GLY A 291 VAL A 292 VAL A 293 THR B 334 ASN B 337 PHE B 377 GLU B 378 PRO B 380 MET B 386 HOH B 623 |
| **11a** | IL-2 | -8.1 | ARG A 38 ASP B 6 PRO B 7 PHE B 15 LYS B 16 THR B 115 GLU B 116 ARG B 117 ILE B 118 TYR B 119 PHE B 121 |
|  | IL-6 | -7.4 | LEU A 93 GLU A 96 VAL A 97 GLU A 100 GLN A 103 GLN A 117 LYS A 121 ILE A 124 THR A 139 PRO A 142 ASN A 145 ALA A 146 LEU A 149 |
|  | COX-2 | -10.6 | GLN A 543 LYS A 546 CYS B 36 ASN B 39 PRO B 40 CYS B 41 GLN B 42 ARG B 44 GLY B 45 GLU B 46 CYS B 47 ASP B 125 THR B 129 TYR B 130 GLY B 135 LYS B 137 ALA B 151 LEU B 152 PRO B 153 GLN B 461 GLU B 465 LYS B 468 ARG B 469 |
|  | Caspase-3 | -7.1 | THR A 62 SER A 63 SER A 65 TYR A 204 TRP A 206 ARG A 207 ASN A 208 SER A 209 TRP A 214 GLU A 248 SER A 249 PHE A 250 SER A 251 PHE A 256 HOH A 645 HOH A 684 HOH A 696 HOH A 733 HOH A 736 |
|  | Caspase-8 | -6.5 | THR A 267 LYS A 268 PRO A 271 LYS A 274 ILE B 318 LYS B 319 LYS B 320 ALA B 321 HIS B 322 ILE B 323 HOH A 621 |
| **12a** | IL-2 | -7.0 | ARG A 38 ASP B 6 PRO B 7 PRO B 8 GLU B 9 ILE B 10 ALA B 13 THR B 14 PHE B 15 THR B 115 ILE B 118 TYR B 119 PHE B 121 TYR B 129 |
|  | IL-6 | -7.9 | ASN A 62 LEU A 63 ASN A 64 LEU A 65 PRO A 66 LYS A 67 MET A 68 LEU A 166 ARG A 169 SER A 170 GLU A 173 PHE A 174 SER A 177 |
|  | COX-2 | -10.8 | ASN A 34 CYS A 36 CYS A 37 ASN A 39 PRO A 40 CYS A 41 GLN A 42 GLU A 46 CYS A 47 TYR A 130 GLY A 135 TYR A 136 LYS A 137 LEU A 152 PRO A 153 PRO A 154 VAL A 155 ALA A 156 GLN A 461 GLU A 465 |
|  | Caspase-3 | -7.2 | SER A 65 TYR A 204 TRP A 206 ARG A 207 ASN A 208 SER A 209 TRP A 214 SER A 249 PHE A 250 SER A 251 ASP A 253 PHE A 256 HOH A 645 HOH A 665 HOH A 684 HOH A 696 HOH A 736 |
|  | Caspase-8 | -7.3 | LEU A 138 ALA A 141 GLN A 142 TRP A 145 ILE A 155 MET A 156 ASP A 157 PHE B 401 HIS B 404 |
| **13a** | IL-2 | -8.5 | ARG A 38 ASP B 6 PRO B 7 PHE B 15 THR B 115 GLU B 116 ARG B 117 ILE B 118 TYR B 119 PHE B 121 |
|  | IL-6 | -7.6 | GLU A 43 THR A 44 LYS A 47 SER A 48 LEU A 102 ARG A 105 PHE A 106 GLU A 107 SER A 108 ASP A 161 THR A 164 |
|  | COX-2 | -10.6 | ASN B 34 CYS B 36 CYS B 37 ASN B 39 PRO B 40 CYS B 41 GLU B 46 CYS B 47 TYR B 130 GLY B 135 TYR B 136 LYS B 137 LEU B 152 PRO B 153 PRO B 154 VAL B 155 ALA B 156 GLN B 461 |
|  | Caspase-3 | -7.3 | SER A 65 TYR A 204 TRP A 206 ARG A 207 ASN A 208 SER A 209 TRP A 214 SER A 249 PHE A 250 SER A 251 ASP A 253 PHE A 256 HOH A 645 HOH A 665 HOH A 684 HOH A 696 HOH A 736 |
|  | Caspase-8 | -7.8 | ALA A 141 TRP A 145 PRO A 154 ILE A 155 MET A 156 ASP A 157 PHE B 401 HIS B 404 |
| **14a** | IL-2 | -8.7 | ARG A 38 ASP B 6 PRO B 7 PHE B 15 LYS B 16 THR B 115 GLU B 116 ARG B 117 ILE B 118 TYR B 119 PHE B 121 |
|  | IL-6 | -8.4 | ASN A 62 LEU A 63 ASN A 64 LEU A 65 PRO A 66 LYS A 67 MET A 68 LEU A 166 ARG A 169 SER A 170 GLU A 173 PHE A 174 |
|  | COX-2 | -10.6 | CYS D 36 CYS D 37 SER D 38 ASN D 39 PRO D 40 CYS D 41 GLY D 45 GLU D 46 CYS D 47 MET D 48 TYR D 130 GLY D 135 TYR D 136 LYS D 137 PRO D 153 PRO D 154 VAL D 155 ALA D 156 GLN D 461 |
|  | Caspase-3 | -7.4 | THR A 62 SER A 63 ARG A 64 SER A 65 TYR A 204 TRP A 206 ARG A 207 SER A 209 SER A 249 PHE A 250 SER A 251 PHE A 256 HOH A 645 HOH A 665 HOH A 684 HOH A 736 |
|  | Caspase-8 | -8.2 | LEU A 138 ALA A 141 GLN A 142 TRP A 145 ILE A 155 MET A 156 ASP A 157 PHE B 401 HIS B 404 |
| **15a** | IL-2 | -7.1 | ARG A 38 ASP B 6 PRO B 7 GLU B 9 ILE B 10 ALA B 13 THR B 14 PHE B 15 GLU B 116 ARG B 117 ILE B 118 TYR B 119 |
|  | IL-6 | -7.4 | GLU A 43 THR A 44 LYS A 47 SER A 48 LEU A 102 ARG A 105 PHE A 106 GLU A 107 SER A 108 ASP A 161 THR A 164 |
|  | COX-2 | -9.1 | PRO C 127 PRO C 128 PHE C 142 LEU C 145 GLN C 374 ASN C 375 ARG C 376 LEU D 145 GLY D 225 HIS D 226 GLY D 227 TYR D 373 GLN D 374 ASN D 375 ARG D 376 GLY D 536 ASN D 537 PRO D 538 |
|  | Caspase-3 | -6.6 | ARG A 75 ARG A 79 LYS A 82 TYR A 83 GLU A 84 VAL A 85 HOH A 617 HOH A 685 HOH A 716 HOH A 722 |
|  | Caspase-8 | -8.2 | LEU A 138 ALA A 141 GLN A 142 TRP A 145 ILE A 152 PRO A 154 ILE A 155 MET A 156 ASP A 157 LYS A 158 PHE B 401 HIS B 404 |
| **16a** | IL-2 | -8.8 | ARG A 38 THR A 41 ASP B 6 PRO B 7 PHE B 15 LYS B 16 THR B 115 GLU B 116 ARG B 117 ILE B 118 TYR B 119 PHE B 121 |
|  | IL-6 | -7.3 | LEU A 93 GLU A 94 GLU A 96 VAL A 97 GLU A 100 LYS A 121 THR A 139 PRO A 140 PRO A 142 ASN A 145 ALA A 146 LEU A 149 |
|  | COX-2 | -10.5 | CYS A 36 ASN A 39 PRO A 40 CYS A 41 GLN A 42 ARG A 44 GLY A 45 GLU A 46 CYS A 47 TYR A 130 LEU A 152 PRO A 153 GLN A 461 GLU A 465 LYS A 468 ARG A 469 |
|  | Caspase-3 | -6.5 | ARG A 75 ARG A 79 LYS A 82 TYR A 83 GLU A 84 VAL A 85 |
|  | Caspase-8 | -6.8 | HIS A 237 CYS A 285 ASP A 288 PRO A 290 VAL B 338 SER B 339 TRP B 340 ARG B 341 HOH A 703 |
